# Supplementary material for: Lipoprotein-biomimetic nanostructure enables efficient targeting delivery of siRNA to Ras-activated glioblastoma cells via macropinocytosis
Source: Nat Commun. 2017 May 10;8:15144. doi: 10.1038/ncomms15144 (PMC5436231; doi:10.1038/ncomms15144)
Supplement: Supplementary Information — Supplementary Figures and Supplementary Table [file ncomms15144-s1.pdf]

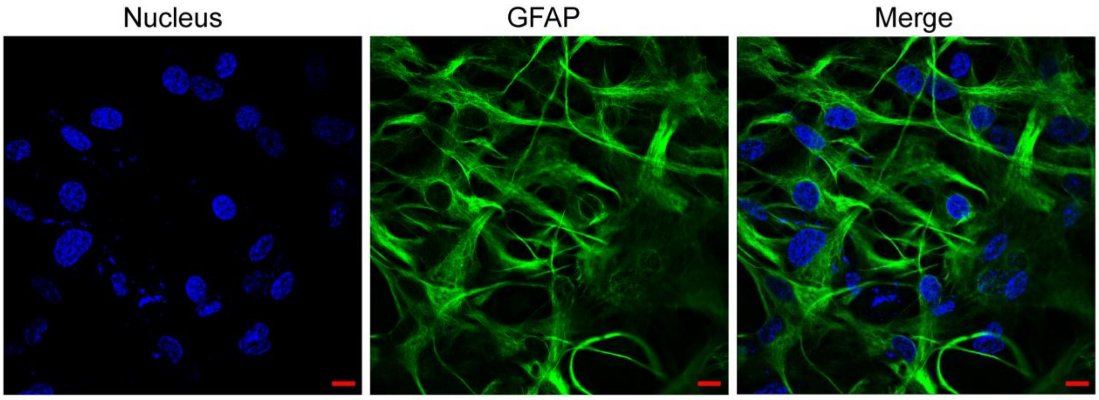

Supplementary Figure 1. Validation of astrocytes. Primary astrocytes were separated from the glial cultures using a mild trypsinization protocol. Anti-glial fibrillary acidic protein (GFAP) immunofluorescent analysis was performed for cell characterization, confirming that the obtained cells were astrocytes. Scale bar, 10 μm.

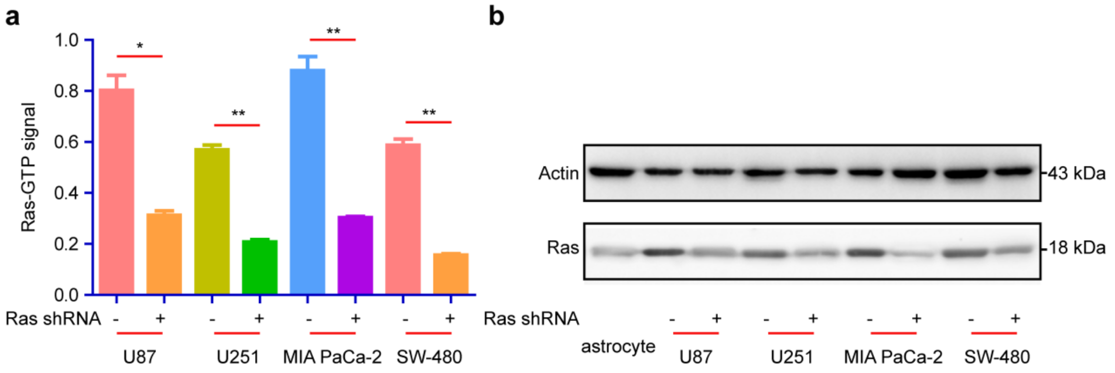

Supplementary Figure 2. Interference RNA-mediated knockdown of Ras in U87, U251, MIA PaCa-2 and SW-480 cells. General all Ras knockdown was accomplished following the application of a short hairpin RNA (shRNA) expression lentivirus system. Ras-GTP **(a)** and Ras protein **(b)** levels in different cell lines before (-) and after (+) Ras knockdown were determined *via* a G-LISA kit and immunoblotting with Ras-specific antibody using actin as the loading control. Data represent mean ± s.d. (n=3). *p< 0.05, **p< 0.01 represent significantly different, Student's t-test (two-tailed).

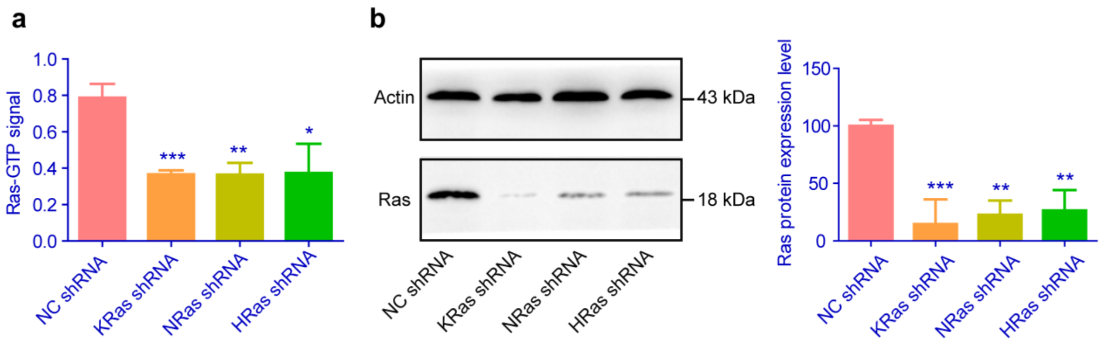

Supplementary Figure 3. Interference RNA-mediated knockdown of Ras in C6 cells. Ras knockdown was achieved following the application of short hairpin RNA (shRNA) expression lentivirus systems. The Ras-GTP **(a)** and Ras protein **(b)** levels in C6 cells transfected with Ras-specific shRNAs (KRas shRNA, NRas shRNA and HRas shRNA) were both detected. C6 cells transfected with a negative control scramble shRNA (NC shRNA) were used as the control group. Data represent mean ± s.d. (n=3). *p< 0.05, **p< 0.01, ***p< 0.001 significantly different with that of the NC-shRNA group, Student's t-test (two-tailed).

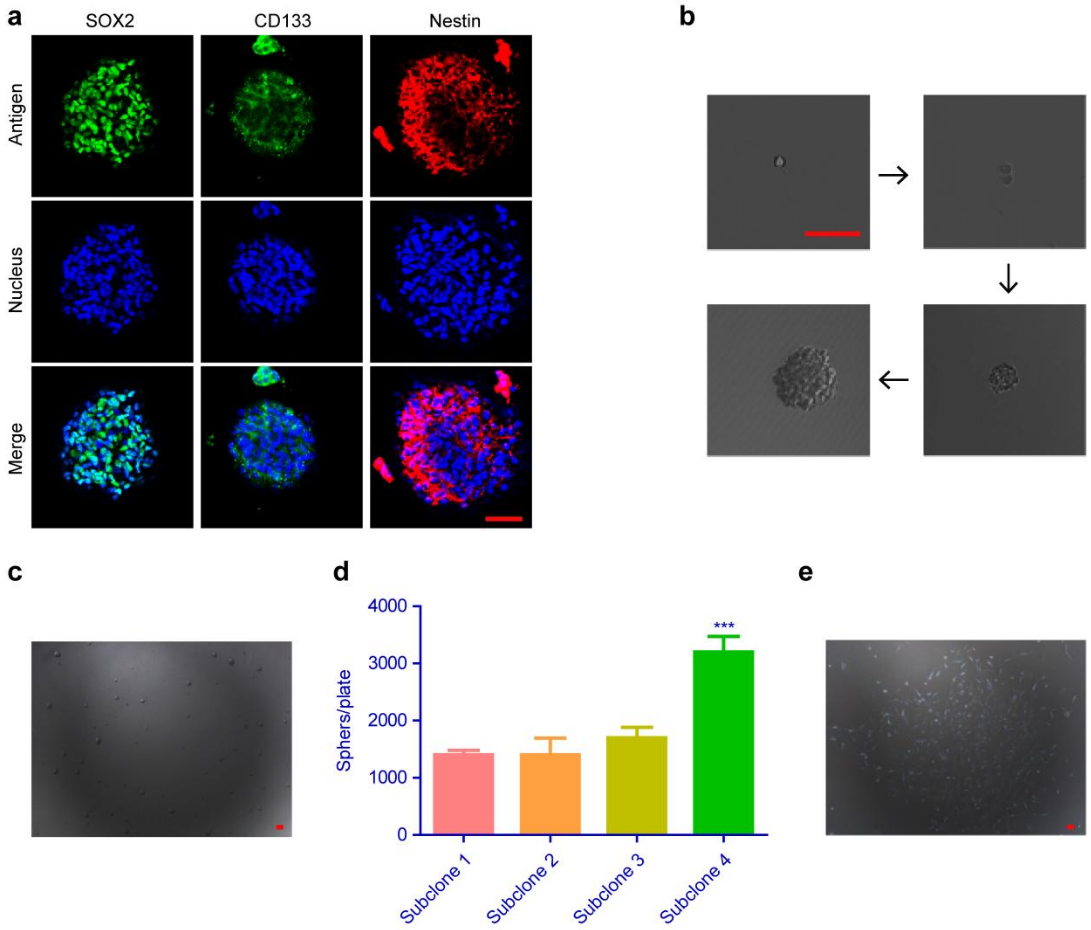

Supplementary Figure 4. Validation of glioblastoma-initiating cells (GICs). (a) GICs spheres derived from patient glioblastoma sample were immunofluorecently stained with anti-SOX2 (green), anti-CD133 (green) and anti-Nestin (red) antibodies. Cell nuclei were counter stained with DAPI (blue). Scale bar, 50 μm. **(b)** The primary GICs spheres were passaged for 10 passages after isolation from patient tumor tissue and then subjected to limiting dilution assay at the density of 0.5 cells per well. The morphology of clone on Day 0 to Day 7 was shown to confirm the self-renewal ability of the cells. Scale bar, 25μm. **(c)** The secondary spheres were generated and passaged, and showed high spheres formation efficiency. Scale bar, 25μm. **(d)** The four subclones generated after the limiting dilution assay were passaged for 3 times to determine the growing ability by counting the number of the spheres, and significant

difference was found among the four subclones. The significance of the differences was evaluated by one way ANOVA followed by Bonferroni test.***$p < 0.001$, significantly different from other subclones. **(e)** The spheres were cultured in DMEM/F12 medium with 10% FBS to test the differentiation ability. Scale bar, 25μm.

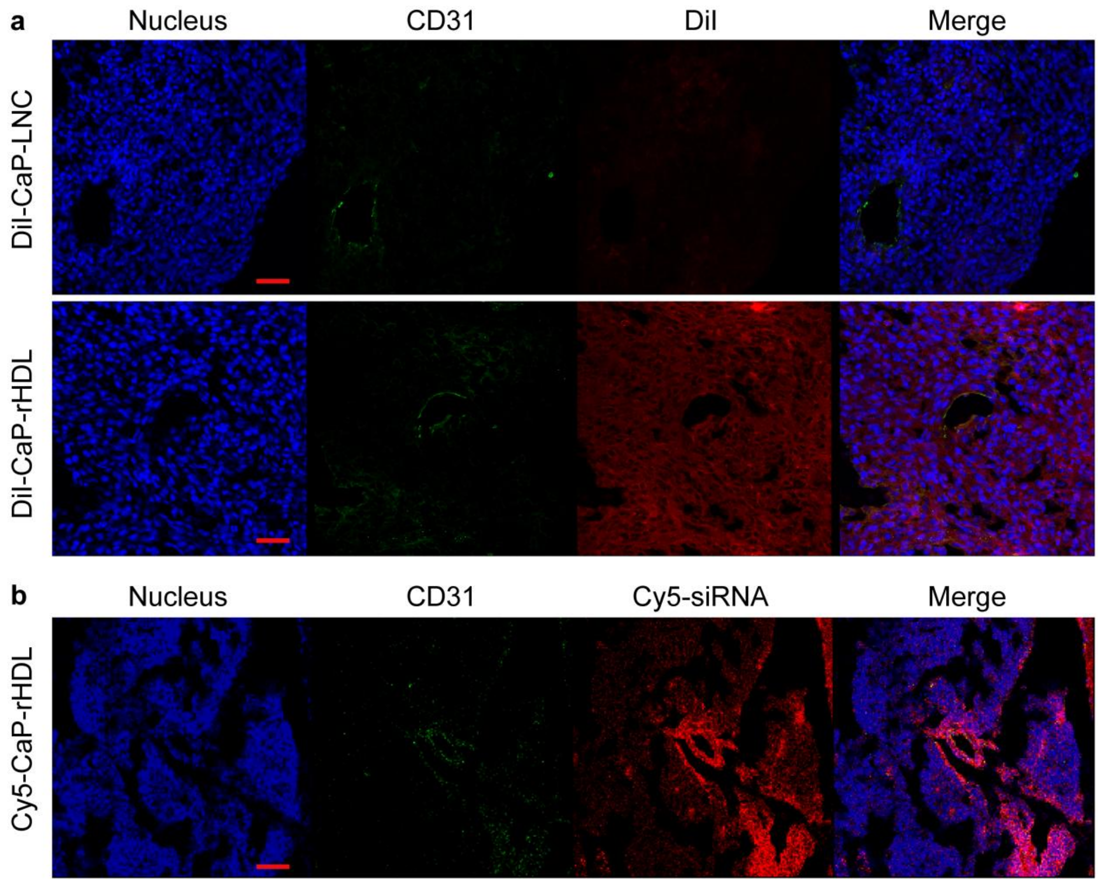

Supplementary Figure 5. Distribution of DiI-CaP-LNC, DiI-CaP-rHDL and Cy5-CaP-rHDL (red) in the glioblastoma sites. Mice bearing intracranial C6 **(a)** or GICs **(b)** glioblastoma were intravenously injected with DiI-CaP-LNC, DiI-CaP-rHDL or Cy5-CaP-rHDL. Three hours later, the animals were euthanized with the brains collected, sectioned and stained before the examination under a confocal microscope. Blood vessels were visualized with anti-CD31 (green), and cell nuclei were stained with DAPI (blue). Scale bar, 100μm.

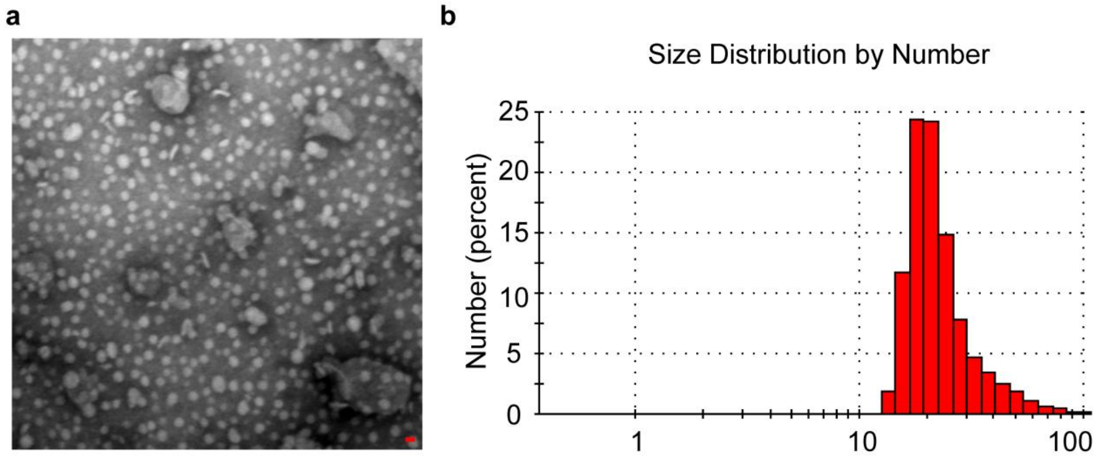

Supplementary Figure 6. Characterization of Cy5-CaP-LNC-PEG. (a) Morphology and particle size of Cy5-CaP-LNC-PEG under a transmission electron microscope after negative staining with sodium phosphotungstate solution (1.75%, w/v). Scale bar, 20 nm. **(b)** Particle size distribution of Cy5-CaP-LNC-PEG analyzed by dynamic light scattering *via* a Zetasizer.

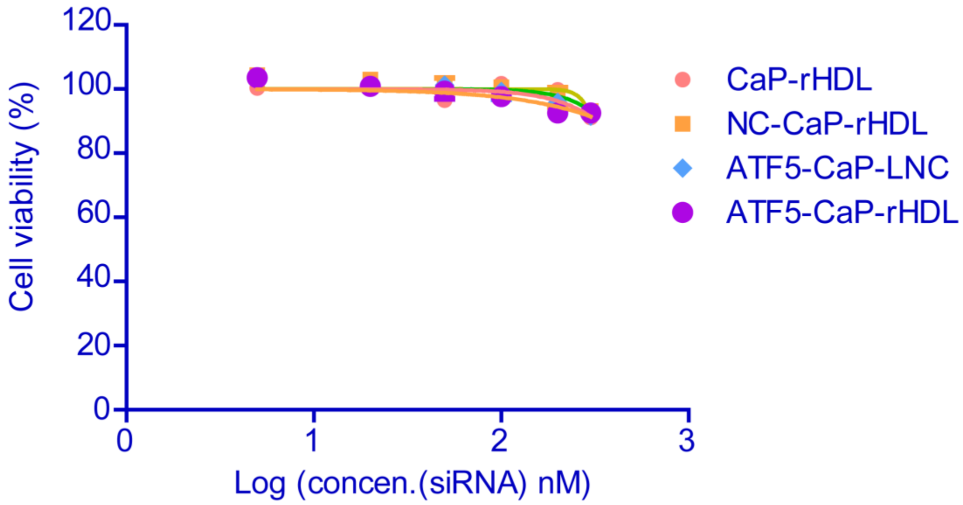

Supplementary Figure 7. Effect of ATF5-CaP-rHDL on the viability of astrocytes. Primary astrocytes were incubated with blank CaP-rHDL, NC-CaP-rHDL, ATF5-CaP-LNC or ATF5-CaP-rHDLat 37°C for 48 h, and the cell viability were evaluated by CCK8 assay (*n*=3).

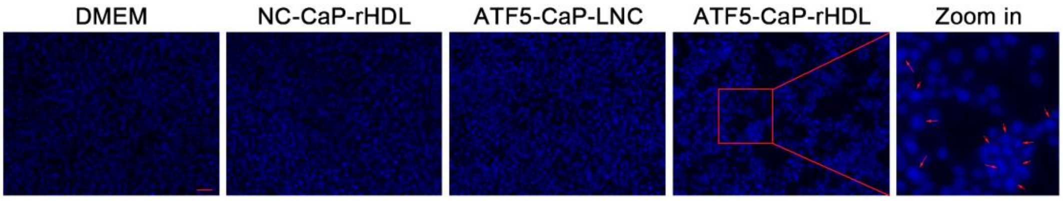

Supplementary Figure 8. ATF5-CaP-rHDL induced apoptosis in C6 cells. C6 glioblastoma cells were incubated with NC-CaP-rHDL, ATF5-CaP-LNC or ATF5-CaP-rHDL at the siRNA concentrations 100 nM for 48 h. Cell nuclei were stained with Hoechst 33342. Red arrows indicate nuclei hyperchromatism in the apoptotic cells. Scale bar, 50 μm.

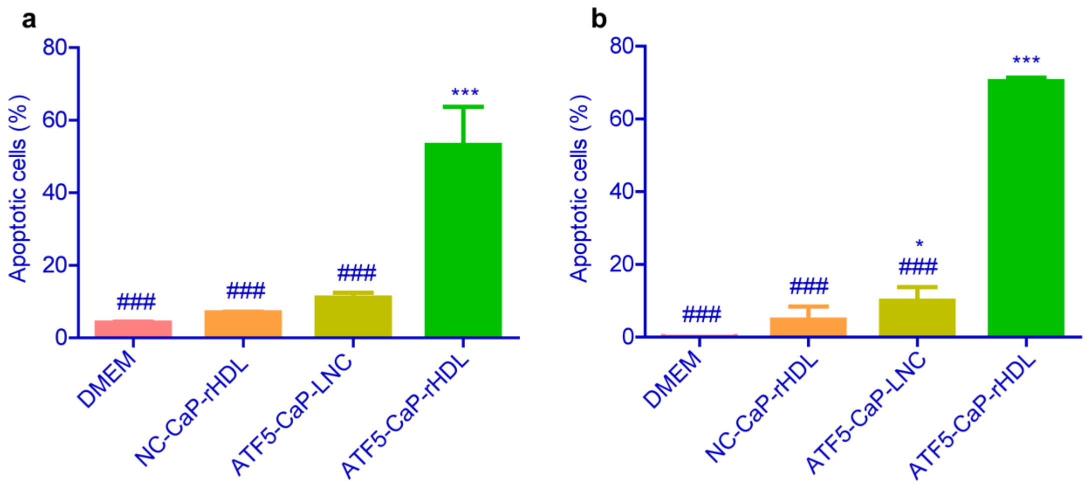

Supplementary Figure 9.ATF5-CaP-rHDL induced the highest extent of apoptosis in C6 cells (a) and GICs (b). C6 cells and GICs were incubated with various siRNA formulations at the siRNA concentrations 100 nM and 200 nM, respectively. Quantitative flow cytometry analysis was performed after staining the cells with Annexin V-FITC and PI. Data represent mean ± s.d. (*n*=3). The significance of the differences was evaluated by one way ANOVA followed by Bonferroni test. **p*< 0.05, ****p*< 0.001 significantly different with that of the DMEM, ###*p*< 0.001 significantly different with that of the ATF5-CaP-rHDL.

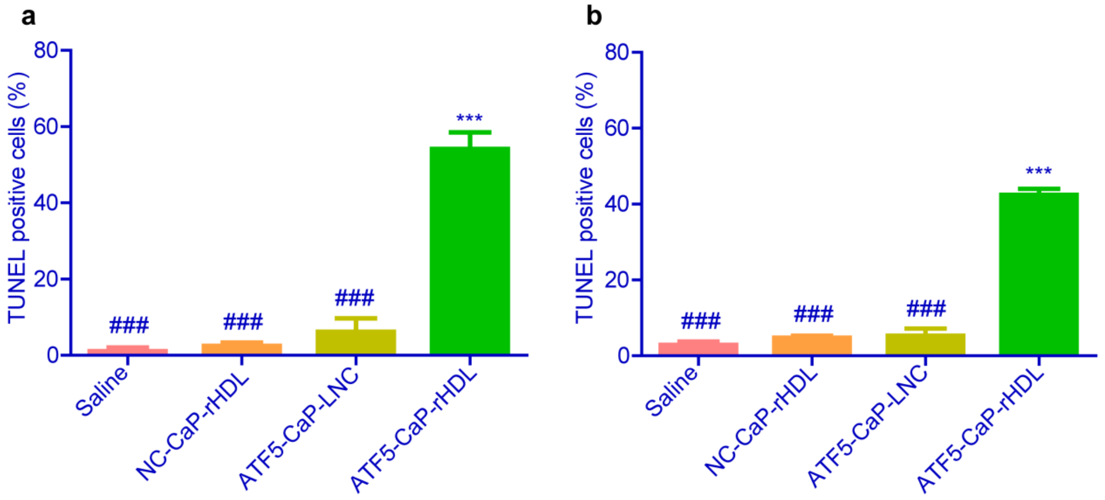

Supplementary Figure 10. ATF5-CaP-rHDL induced severeapoptosis at the glioblastoma sites in both mice models bearing intracranial glioblastoma. The mice bearing C6 **(a)** or GICs **(b)** intracranial glioblastoma were treated with saline, NC-CaP-rHDL, ATF5-CaP-LNC or ATF5-CaP-rHDL for four or five times at siRNA dose of 0.36 mg kg^{-1} (n = 3). One day after the last injection, the brains were collected, sectioned and stained using a TUNEL (tdt-mediated dUTP nick-end labeling) kit. For image-based quantification, at least 200 cells were scored per experiment. Data represent mean±s.d. (n=3). The significance of the differences was evaluated by one way ANOVA followed by Bonferroni test. ***p< 0.001 significantly different with that of the saline, $^{###}p$< 0.001 significantly different with that of the ATF5-CaP-rHDL.

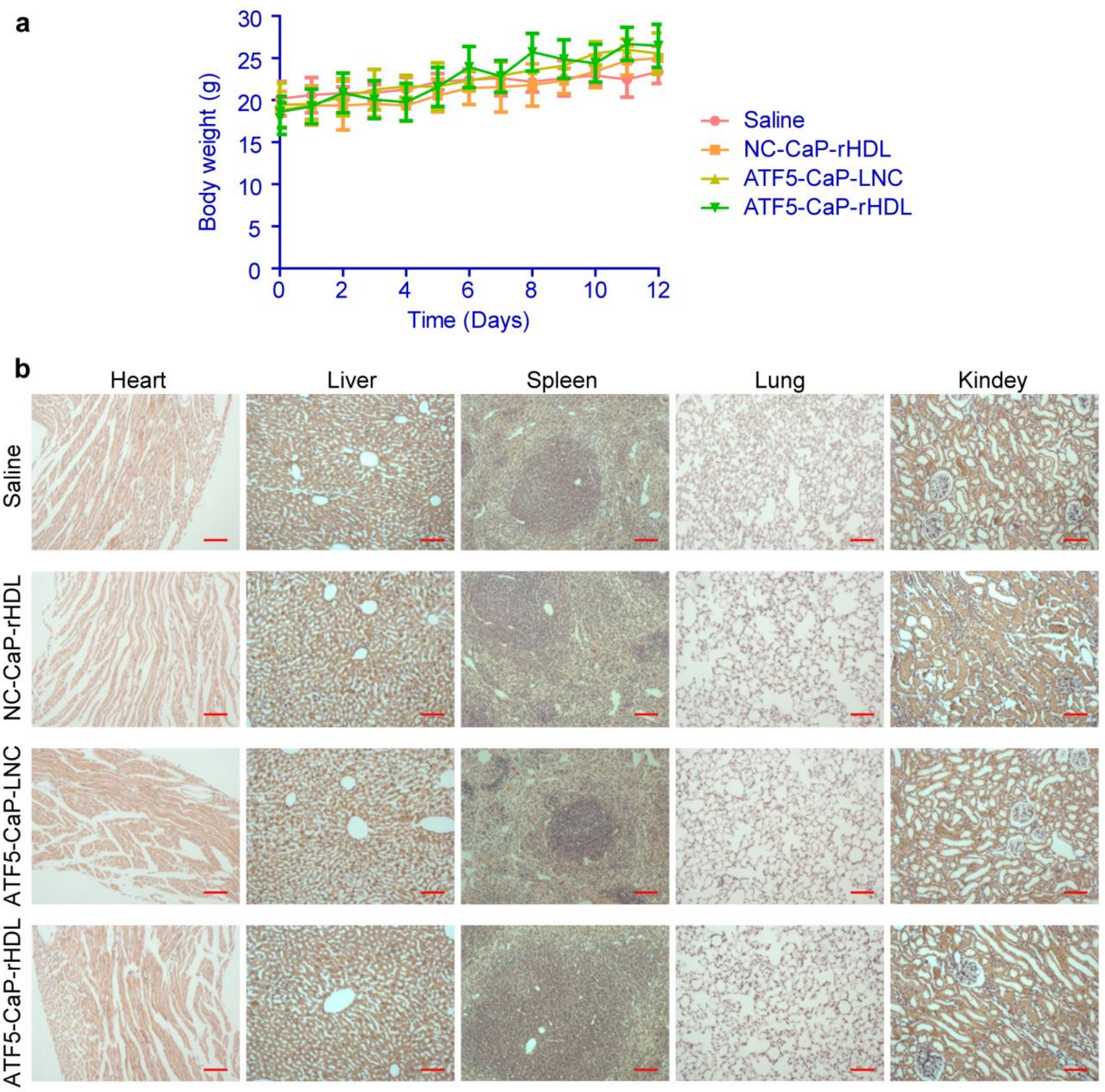

Supplementary Figure 11.ATF5-CaP-rHDL did not cause observable side effects in nude mice bearing C6 glioblastoma. (a) The body weight curve of the model animals following the treatment with saline, NC-CaP-rHDL, ATF5-CaP-LNC or ATF5-CaP-rHDL every two days for four times at siRNA dose of 0.36 mg kg^{-1} (n = 9). **(b)** Hematoxylin and eosin staining of the major organs (heart, liver, spleen, lung, kidney) from the model mice following the treatment with saline, NC-CaP-rHDL, ATF5-CaP-LNC or ATF5-CaP-rHDL every two days for four times at siRNA dose of 0.36 mg kg^{-1} (n = 9). Scale bar, 100 μm.

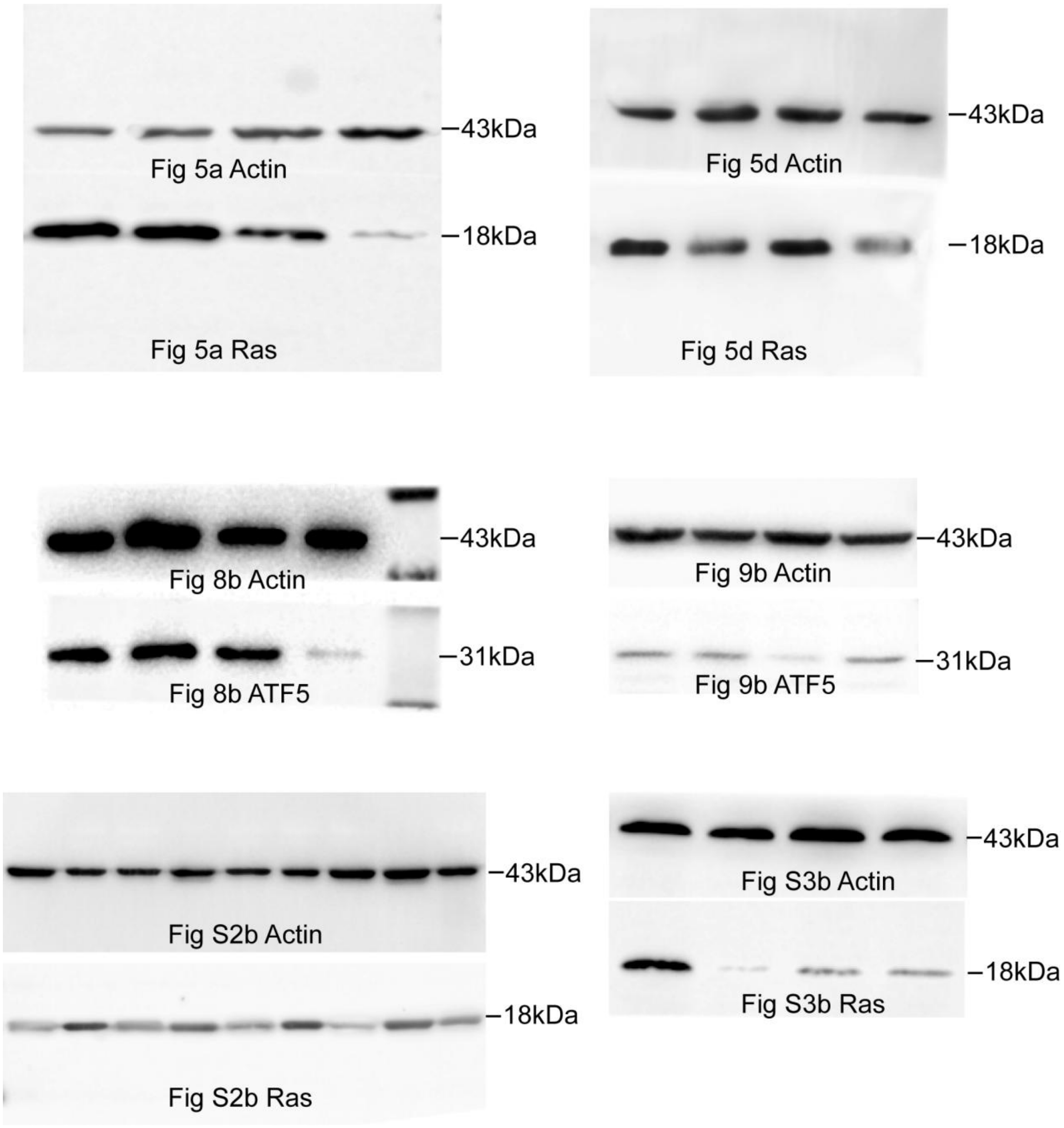

Supplementary Figure 12.Original western blots for the images used in Fig.5, Fig.8, Fig.9, Fig.S2 and Fig.S3

	Particle size (nm)	Polydispersity index	Zeta potential (mV)
**CaP-LNC**	30.79 ±1.49	0.39	−15.90 ±1.39
**CaP-rHDL**	24.04 ±8.42	0.29	−22.33 ±0.42
**DiI-CaP-LNC**	24.54 ±2.92	0.34	−16.27 ±1.30
**DiI-CaP-rHDL**	24.59 ±8.40	0.27	−23.73 ±0.99
**DiR-CaP-LNC**	34.83 ±2.53	0.39	−9.67 ±0.87
**DiR-CaP-rHDL**	28.3 ±10.78	0.33	−18.03 ±1.65
**FAM-CaP-LNC**	27.60 ±0.64	0.30	−17.87 ±1.80
**FAM-CaP-rHDL**	29.14 ±1.88	0.26	−24.63 ±3.58
**Cy5-CaP-LNC-PEG**	24.36 ±2.18	0.28	−23.90 ±2.65
**Cy5-CaP-rHDL**	28.05 ±3.20	0.29	−25.30 ±3.14
**NC-CaP-LNC**	23.57 ±3.17	0.35	−16.27 ±1.30
**NC-CaP-rHDL**	22.67 ±4.8	0.31	−26.33 ±1.51
**ATF5-CaP-LNC**	27.21 ±2.94	0.28	−18.53 ±1.53
**ATF5-CaP-rHDL**	30.57 ±8.97	0.25	−24.00 ±2.81

Supplementary Table 1. Particle size and zeta potential of nanoparticles.
